# Nurses’ Work–Family Strategies during COVID-19 Lockdown and Their Association with Individual Health and Family Relations

**DOI:** 10.3390/healthcare11222960

**Published:** 2023-11-14

**Authors:** Jia-Lin Zhao, Li Shen, John Shields, Ya-Xuan Wang, Yu-Jia Wu, Zhan Yu, Yi-Xin Li

**Affiliations:** 1 Department of Sociology, College of Philosophy, Law and Political Science, Shanghai Normal University, Shanghai 200234, China; zhaojialin@shnu.edu.cn (J.-L.Z.); 1000512959@smail.shnu.edu.cn (Y.-X.W.); 1000528636@smail.shnu.edu.cn (Y.-J.W.); 1000501468@smail.shnu.edu.cn (Y.-X.L.); 2 Department of Sociology, School of Public Affairs, Nanjing University of Science and Technology, Nanjing 210014, China; 3 Discipline of Work and Organisational Studies, The University of Sydney Business School, The University of Sydney, Sydney, NSW 2006, Australia; john.shields@sydney.edu.au; 4 Department of Social Work, School of Social Development, East China Normal University, Shanghai 200062, China; yz@stu.ecnu.edu.cn; 5 Shanghai Social Science Innovation Research Base of “Research on Transitional Sociology with Chinese Characteristics”, Shanghai 200062, China

**Keywords:** nurses, childcare, COVID-19, quarantine, mental health, family relations, work performance

## Abstract

The COVID-19 lockdown forced people to stay at home and address their family duties more equally. However, since nurses themselves were involved in the closed-loop management in hospitals and unable to return home, there was also an increased likelihood of non-traditional work-family strategies emerging. To ascertain the extant and implications of this phenomenon, this cross-sectional study explores work–family management strategies among nurses during the COVID-19 lockdown and their association with nurses’ individual health, family relationships, and job performance. Survey data were collected from 287 nurses who were involved in the closed-loop management in Shanghai hospitals from March to June 2022. Latent Class Analysis of seven categorical variables of nurses’ work–family status (e.g., the division of childcare labor) produced a best-fit solution of five strategies (BLRT (*p*) < 0.001, LMR (*p*) = 0.79, AIC = 5611.34, BIC = 6302.39, SSA-BIC = 5703.65, Entropy = 0.938): (1) fully outsourcing to grandparents, (2) partially outsourcing to grandparents, with the husband filling in the gap, (3) the husband does it all, (4) egalitarian remote workers, and (5) a neo-traditional strategy. Nurses who applied the egalitarian strategy had less psychological distress and relationship tension and better performance than those who applied the neo-traditional strategy and performed most of the childcare. The “husband does it all” strategy and the outsourcing strategies seem to have double-edged effects, with better job performance and family relations but also more distress and fewer sleeping hours among nurses. Overall, with a view to future risk mitigation, policymakers and practitioners should be aware of the diversity of the work–family strategies among nurse families during the lockdown period, and their association with individual and family outcomes, and provide tailored support.

## 1. Introduction

Nurses made a remarkable contribution to public health across many countries during the COVID-19 pandemic, but the evidence now emerging shows that they also experienced various personal and family health problems [1,2,3]. Recent research has found that the pandemic affected people’s physical and mental health and family relationships via forced changes to their work–family management strategies [4]. Work–family management strategies are often highly gendered, with women taking on more childcare responsibilities and men focusing on their jobs [5,6]. Although the division moved toward more gender equality during the pandemic of COVID-19 due to the remote work status of both genders, women still performed more childcare and housework than men [7,8,9,10,11,12]. The situation was more challenging for female health workers, particularly nurses. Due to the lack of childcare support and the closure of schools during the pandemic, nurses struggled to fulfill both their work and family roles [13,14]. Their childcare responsibility reduced their participation in work [13,15,16,17] and further led to burnout and depression [16,18,19,20,21].

These challenges became most acute during the COVID-19 lockdown in Shanghai from early March to early June 2022, when the lockdown was protracted and strictly enforced. At that period, many hospitals were in a closed-loop management situation [22,23]. Nurses were required to undertake a large number of prevention and management tasks and were unable to return home due to the policies that restricted movement and completely closed the local communities [24,25]. In other words, because of the spread of the pandemic and the “Zero Covid” policy, all the facilities and local communities were gradually and completely closed with no one moving in or out. These restrictions may have further disrupted nurses’ pre-existing work–family management strategies, forcing them to adopt alternative approaches, including their husbands spending more time and energy on family duties [26,27] and the involvement of other relatives, mainly grandparents, in childcare [28,29]. It is also important to assess and compare the impact of these alternative strategies on nurses’ physical and mental health, family relationships, and job performance.

Overall, the COVID-19 lockdown in Shanghai from early March to early June 2022 can be viewed as a socially-engineered experiment reinforcing the emergence of more gender-equal or even gender-asymmetrical work–family strategies and more outsourcing strategies among nurse families. Such changes in work–family strategies are also likely to influence the personal and family outcomes of nurses. Accordingly, this study has two main objectives. The first is to describe and classify the main work–family management strategies used by nurse families during the Shanghai lockdown. The second objective is to explore the associations of these strategies with nurses’ physical and mental health, family relationships, and job performance. We restricted our exploration to female nurses and heterosexual couples because work–family management strategies have traditionally been highly gendered, with women taking on more family duties [5].

## 2. Research Questions

Work–family management strategies are the methods, plans, or actions for managing work and family roles [30]. Childcare is often the focus of those strategies. The expectations before the birth of a child and the subsequent division of housework can influence the well-being of spouses and their family relationships [31,32]. A traditional work–family management strategy includes men working outside and women staying at home to perform all household work and childcare [5,33]. Long-term trends in the gender division of labor and the rising cost of family formation and growth have increased the prevalence of dual-career families, with male partners in paid employment also undertaking more tasks at home, while women have increased their participation in paid employment outside of the home [33]. Neo-traditional work–family management strategies have emerged in dual-career families, with women preferring more flexible work to meet their family roles and men focusing on work to fulfill their role expectations [6,34]. However, these neo-traditional approaches are still unequal for women. Women who work more than 35 h per week also take on many household tasks, especially childcare [35]. Likewise, women still perform the majority of household work in Chinese families [36].

The COVID-19 pandemic has blurred the line between work and family. Shockley et al. found that spouses adopted an egalitarian strategy by shifting their working days and schedules to fulfill their work and family roles [4]. Men also took on more family work during this period, particularly when they were in the status of remote work [26,27,37]. Other family members, such as grandparents, and outside helpers, also took on more family responsibilities during the lockdown period [29,38]. Nevertheless, women still took on the main responsibilities and performed more childcare and housework than men [7,8,9,10,11,12,39,40,41]. In particular, nurses struggled to balance both their work and family roles, which became a major barrier for them to participate in the work of healthcare during the pandemic [13,14,15].

The situation involved in this study is much more severe than the typical pandemic lockdown. During the Shanghai lockdown period (March to June 2022), a large number of nurses worked in hospitals with closed-loop management and were not able to return to their families at all due to the lockdown policy, which restricted movement and forced closure of all facilities [24,25]. In such an extreme lockdown situation, nurses’ husbands who were forced to stay at home due to the policies may have taken on the main duties of household and childcare [26,27], which may have led to more gender equality in household work or even a reversed gender-asymmetrical pattern of work–family strategies. It is also intriguing to examine if grandparents became the major stable source of outside help during the prolonged lockdown period. Grandparenting is not common in Western cultures; it usually exists in families with disruptive events, such as parents’ divorce or parental death [42,43]. In contrast, nearly half of Chinese families are extended families with grandparents living together [42]. The Confucian teaching of familism, which emphasizes the connection of family members and family duties, also had a great impact on Chinese families [43]. Many Chinese grandparents even substitute the main role of motherhood in taking care of their young grandchildren [44]. Accordingly, it is possible that outsourcing household work and childcare to grandparents may have become a main work–family strategy among nurses who worked in hospitals and could not return home during the lockdown period. To clarify these possibilities, we propose the following research question:

Research Question 1: *What were the work–family management strategies of nurse families during the COVID-19 lockdown?*

Further, traditional work–family strategies usually have negative effects on women’s physical and mental health, family relationships, and job performance, while more gender-balanced strategies have positive effects. Large differences in hours of household work affect women’s mental health [45] and their relationship satisfaction [46]. A more balanced division of housework between couples not only has positive effects on women’s physical and mental health but also improves their male partners’ happiness [32]. In addition, more time spent by men in childcare helps reduce women’s work stress [47].

During the pandemic, families using neo-traditional strategies also had lower levels of family cohesion, family relationships, and job performance than those with an equal division of household work [4]. Although men spent more time in household work and childcare, women were still exhausted from fulfilling both their motherhood and work duties, with less sleep time, more mental health problems, and more relationship tensions [12,39,48,49].

Past studies have not focused on nurses in extreme lockdown situations. If nurses could not return home due to the closed-loop management in hospitals and the lockdown policy, their husbands and other family members (e.g., grandparents) may have had to assume the main family roles and even perform all household work. The involvement of fathers and grandparents may increase mothers’ participation in the workforce [43,50]. Although previous research suggests the benefits of men’s participation in household work [47], a sudden transfer to a completely gender-reversed strategy may lead to role ambiguity and stress on both sides [48,51,52,53]. There is also evidence that long-term grandparenting can have both positive and negative effects on children and their carers (e.g., emotional and behavioral dysfunction of grandchildren) [43]. To further explore the association between these non-traditional strategies and nurses’ individual and family outcomes, we follow Shockley et al. [4] and propose a second research question, as follows:

Research Question 2: *To what extent were the work–family management strategies during the COVID-19 lockdown related to nurses’ physical and mental health, family relationships, and job performance?*

## 3. Materials and Methods

### 3.1. Research Design

This cross-sectional study describes the work–family strategies among nurse families during the COVID-19 lockdown and further explores their association with nurses’ individual health, family relations, and job performance. This study was based on a survey of 287 nurses who experienced the lockdown period in Shanghai. We used convenience sampling to collect the survey data. We further used Latent Class Analysis (LCA) to classify work–family management strategies and then explored their association with nurses’ individual and family outcomes.

### 3.2. Participants and Procedure

The survey data were collected in August 2022. We selected seven tertiary hospitals in Shanghai (all of which were in closed-loop management from March to June 2022) to collect data. The sampling method was convenient sampling. First, we contacted the head nurses at the hospitals and asked for permission to conduct the study. The head nurses then helped us send the link of online survey to the nurses. The survey was built on Wen Juan Xing (wjx.cn), a widely used commercial provider of online survey systems. The survey was not open to the public (password protected). We also asked the head nurses to only distribute the survey to the nurses in their hospitals. Participation in this study was wholly anonymous and voluntary. After completing the survey, each participant was given an allowance of CNY 20 (approximately USD 3). The survey only had two pages (excluding the participation information statement). There was a back button on each of the pages. Each participant could only complete the survey once based on their IP addresses and cookies of the website (functions provided by Wen Juan Xing). The average completion time of the participants was 7 min.

Regarding the inclusion and exclusion criteria of the participants, because childcare is often the main focus of work–family strategy [4,31,32], we only included nurses with at least one child under Grade 5. The reason for choosing Grade 5 as a cut-off is that Chinese parents are also heavily involved in childcare and education of their primary school children [54].

We had a total of 330 responses from the online survey. We checked the completeness of the survey manually. After excluding the incomplete responses and those not meeting the criteria (also including one response from a male nurse), the final sample included 287 valid responses, with a completion rate of 87.0%. The sample size was close to the rough range for LCA (N ≈ 300–1000) suggested by Nylund-Gibson and Choi [55] and comparable to that in Shockley et al. [4]. Since we also used robust separating items based on Shockley et al. [4] and had predictions on potential classes, the sample size should be adequate to identify the potential strategies. The nurses were from a wide range of departments in the hospitals, such as emergency, out-patient, ICUs, pneumology, and infectious disease. Most of them worked in the hospitals during the COVID-19 lockdown, with 177 taking care of the patients, and 150 also performing nucleic acid testing at the same time.

### 3.3. Measurement

#### 3.3.1. Independent Variables

We developed our survey mainly based on Shockley et al.’s, which has been proven to be reliable and valid [4]. Seven variables were used to classify work–family management strategies of nurses, including the remote work states of nurses, changes to couples’ shift and work hour arrangements, the division of childcare labor during normal work hours, and the outside help.

The remote work states of nurses. To check if the work status of nurses were similar to Shockley et al.’s measure of work–family strategies [4], we conducted a pilot study by interviewing 13 nurses about their working and family status during the lockdown period. Details of the interviews can be obtained from the first author. Based on the interviews, we found that nurses had four main working states similar to options proposed by Shockley et al. [4]: (1) not working remotely, i.e., working in hospitals for the whole lockdown period and not returning home due to the closed-loop management policy of the hospitals or the complete lockdown of the local communities; (2) working remotely partially according to stable schedules (e.g., working five days in the hospital and two days at home per week by negotiating with both the hospital and the local community); (3) mostly working remotely (e.g., working in hospital at the beginning and then at home all the time due to the later complete lockdown of the local communities); and (4) working remotely full time (staying at home for the whole lockdown period due to the early complete lockdown of the local communities). We used these four options in our survey.

Shift change. Following Shockley et al. [4], we measured shift changes of nurses and their partners during the lockdown period by three options: (1) not changing shifts; (2) informally changing shifts (e.g., working late or early hours); and (3) formally changing a shift.

Amount of work hour change. Following Shockley et al. [4], we measured changes in total work hours of nurses and their husbands by three options: (1) not adjustment hours; (2) reducing hours; and (3) increasing hours.

The division of childcare labor during normal work hours. Following Shockley et al. [4], we measured the division of childcare during the lockdown period with 12 options: (1) performing all childcare during work hours; (2) performing most of the childcare except occasional relief; (3) alternating working days with spouse; (4) staggering shift work (e.g., the husband works at night and the wife takes care of the children); (5) having mini-shifts throughout day while both are remote; (6) both working remotely, alternating childcare with spouse based on meeting commitments; (7) only providing occasional childcare relief; (8) full outsourcing; (9) performing all childcare when it is not outsourced; (10) performing none of the childcare when it is not outsourced; (11) performing none of the childcare; and (12) other division, please specify. The responses to option 12 were further coded and merged into the previous options. Since options 10 and 11 have similar meanings, and LCA only allows a maximum number of 10 options per category [4], we combined the two options to reduce the complexity of the analysis.

Outside help. Following Shockley et al. [4], we used ten options to measure outside help of nurse families during the lockdown period: (1) no outside help; (2) relative, friend, or older sibling helping part-time; (3) relative, friend, or older sibling helping full-time; (4) paid help (e.g., nanny or babysitter) part-time; (5) paid help full-time; and (6) relative/friend helping part-time and paid help part-time. In addition, based on our interviews with nurses, we added four options: (7) part-time help from the elders (e.g., grandparents); (8) full-time help from the elders; (9) part-time help from community social workers or volunteers; and (10) full-time help from community social workers or volunteers.

#### 3.3.2. Dependent Variables

We measured nurses’ physical and mental health, family relationships, and job performance based on assessments from Shockley et al. [4].

Physical and mental health. We measured the health state of nurses during the lockdown period by including two variables: sleep quality and psychological distress. Following Shockley et al. [4], sleep quality was measured by average hours of sleep at night—“during the COVID-19 lockdown in Shanghai from early March to early June 2022, on average, how many hours of actual sleep did you get at night”? We assessed psychological distress via ten items from Kessler et al. [56]. Participants self-rated on a five-point scale. A sample question is as follows: “During this time period, how often have you felt tired out for no reason”? The scale has good reliability and validity among the Chinese population [57]. The Cronbach’s α of the scale was 0.95 in this study.

Family relationship. Family relationship includes two variables: family cohesion and relationship tension. Following Shockley et al. [4], we used three questions from Huffman et al. [58]. A sample item is as follows: “In thinking about your family during this time, family members really helped and supported one another”. We also used three questions from Mathews et al. [59] to measure relationship tension based on a five-point scale. A sample item is as follows: “During this period, I have felt irritated or resentful about things my partner did or didn’t do”. The Cronbach’s α of the two scales was 0.96 and 0.89, respectively.

Job performance. Following Shockley et al. [4], we measured job performance by two methods. First, participants self-rated their performance based on four items from Abramis [60] on a five-point scale. A sample question is as follows: “During this period, how well have you handled the responsibilities and daily demands of your work”? The Cronbach’s α of the scale was 0.93. Then, participants were asked to select the percentage of work responsibilities they performed during the lockdown period on a scale from 0% to 100% to measure relative job performance.

#### 3.3.3. Control Variables

We selected potential control variables based on Shockley et al. [4]: number of children under 18, number of children under Grade 5, monthly household income from 1 “less than 5000 Chinese Yuan” (approximately USD 800) to 10 “more than 100,000 Chinese Yuan” (approximately USD 15,000), the ages of nurses and their husbands, and the age differences of the couples.

### 3.4. Analytical Strategies

Following Shockley et al. [4], we analyzed the data in four steps. First, we described the independent (categorical) variables associated with work–family management strategies during the lockdown period, including the remote work states of nurses, shift changes and amount of work hour changes of the couples, and the division of childcare labor during normal work hours and outside. Second, we described the means and SDs of the control and dependent variables and provided their Pearson correlations. Third, for Research Question 1, we used mplus8.2 to conduct LCA on the independent (categorical) variables to clarify nurses’ work–family management strategies. LCA can identify latent subgroups based on responses from individuals’ multivariate categorical data, which begins with one group and increases the groups until fit is no longer substantially improved [4]. Following the best practice recommendations [61], we determined fit by looking for lower values in the Akaike information criterion (AIC), consistent Akaike information criterion (C-AIC), Bayesian information criterion (BIC), sample-size-adjusted Bayesian information criterion (SSA-BIC), Lo–Mendell–Rubin (LMR) likelihood ratio test, bootstrapped log-likelihood ratio test (BLRT), and Entropy. Fourth, for Research Question 2, we used a three-step robust method [62] to examine the mean differences of the dependent variables among the groups of work–family strategies. In the analysis, the dependent variables were examined in relation to their class membership, taking into account the error in class prediction [62]. Chi-square tests were used to further show the significant differences. Before the analysis, we also examined potential mean differences of the control variables among the groups of work–family strategies. If the control variables have significant mean differences among the groups, we will regress the dependent variables on them and use the residuals in the three-step analysis [63].

## 4. Results

### 4.1. Description of the Independent Categorical Variables of Work–Family Strategies

The description of the independent categorical variables is shown in Table 1. For remote work states, 46.7% of the nurses stayed in hospitals throughout the whole lockdown period due to the early complete lockdown of the hospitals and local communities, and 25.4% worked remotely partially (e.g., five days working and staying in hospitals and two days staying at home by negotiating with both the hospital and the local community). A total of 20.6% of the nurses worked at hospitals at the beginning but stayed at home for the remaining time due to the later complete lockdown of the communities. A total of 60.6% of the nurses had increased working hours during the lockdown, while the majority of their husbands (43.2%) had reduced hours.

In the division of childcare labor, nurses generally took on less responsibility: 25.4% performed none of the childcare, and 15.3% provided only occasional childcare relief. In contrast, their husbands played a pivotal caring role, with 20.2% contributing all childcare during normal work hours and 17.8% undertaking most of the childcare except for occasional relief. Outsourcing childcare (18.5%) was also a main way of dealing with the absence of nurses. Most outside help came from grandparents (full-time help 60.3%; part-time help 10.8%).

### 4.2. Descriptive and Bivariate Analysis of the Control Variables and Nurses’ Health, Family Relationships, and Job Performance

The means and standard deviations of the control and outcome variables and their correlations are shown in Table 2. The average age of the nurses was 36.04 years (SD = 4.43) and that of their husbands was 38.19 years (SD = 5.22). The mean age difference was 2.15 years (SD = 3.32). The mean of household incomes was 4.11 (SD = 1.47) as 28.2% of the participants selected 4 “15,001–20,000 Chinese Yuan” (approximately USD 2000–2800) and 26.5% selected 5 “20,001–30,000 Chinese Yuan” (approximately USD 2800–4100). A total of 88.5% of the nurses had one child, and 11.5% had two children under Grade 5. A total of 82.6% of the nurses had one child, and 17.4% had two children under 18 years old. Considering the relations between the control variables and outcome variables, only husbands’ age was positively related to nurses’ sleep hours (r = −0.12, *p* < 0.05).

### 4.3. Work–Family Management Strategies of Nurses during the COVID-19 Lockdown

For Research Question 1, we conducted an LCA with the seven categorical variables related to work–family management strategies. We examined the indicators based on the best practice recommendations by Gabriel et al. [62] and gave priority to BLRT, LMR, AIC, BIC, and SSA-BIC. The results are shown in Table 3. The solution of five classes (BLRT (*p*) < 0.001, LMR (*p*) = 0.79, AIC = 5611.34, BIC = 6302.39, SSA-BIC = 5703.65, Entropy = 0.938) had the best fit.

Table 4 shows a description of each strategy, including the options with the highest percentages in each variable, which allows us to interpret each specific strategy. More than half of the families chose a reversed gender-asymmetrical strategy or non-traditional strategy [3], such that husbands and other family members were responsible for housework and childcare and nurses were less involved and mainly focused on their hospital work. These strategies included the husband doing it all (29.3%), fully or partially outsourcing to grandparents and the husband filling in the gap (25.8%), and fully outsourcing to grandparents (18.5%). We also found a strategy of egalitarian remote workers with an alternation of work days and hours (20.6%), which is consistent with Shockley et al.’s [2] finding. Finally, a small percentage of families adopted the neo-traditional strategy (5.9%), such that women worked at home and took care of the children. This strategy was mainly adopted by nurses who worked in the hospital at the beginning and subsequently spent most of their time at home.

### 4.4. The Associations of Work–Family Management Strategies with Nurses’ Physical and Mental Health, Family Relationships, and Job Performance

For Research Question 2, we first used the three-step robust method [14] to test the between-group mean differences of the control variables among the strategies and found that only the age of the nurses and their husbands showed statistically significant differences. Therefore, the two were used as control variables, and the residual mean of each dependent variable was calculated by the regression analysis [64]. We further used the three-step robust method to test the between-group mean differences of the dependent variables among work–family management strategies. The results of the Chi-square tests are shown in Table 5.

Regarding physical and mental health, sleep hours showed significant between-group mean differences (χ^2^ = 9.50, *p* < 0.05). The shortest sleep hours were found in the full outsourcing to the grandparents strategy, with a mean of only 6.17 h, lower than the strategy of egalitarian remote workers (χ^2^ = 7.35, *p* < 0.01) and the neo-traditional strategy (χ^2^ = 4.38, *p* < 0.05). Psychological distress also showed significant between-group mean differences (χ^2^ = 14.63, *p* < 0.01). The strategy of fully outsourcing to the grandparents (χ^2^ = 9.31, *p* < 0.01) and the husband doing it all (χ^2^ = 11.55, *p* < 0.001) were higher than the strategy of egalitarian remote workers.

Regarding family relationships, family cohesion showed significant between-group mean differences (χ^2^ = 8.45, *p* < 0.1). The strategy of full outsourcing to grandparents had the highest family cohesion and was higher than the strategy of egalitarian remote workers (χ^2^ = 5.67, *p* < 0.05). Although between-group mean differences in relationship tension were non-significant (χ^2^ = 5.98, *p* > 0.1), relationship tension in families adopting a neo-traditional strategy was statistically significantly higher than that in families choosing all the other strategies.

Finally, in terms of job performance, self-rated general job performance showed significant between-group mean differences (χ^2^ = 8.04, *p* < 0.1). The neo-traditional strategy had the lowest general job performance and was lower than the strategy of full outsourcing to grandparents (χ^2^ = 4.53, *p* < 0.05), the strategy of egalitarian remote workers (χ^2^ = 4.48, *p* < 0.05), and the strategy of fully or partially outsourcing to grandparents and the husband filling in the gap (χ^2^ = 7.05, *p* < 0.01).

## 5. Discussion

The aims of this study were to describe the work–family management strategies of nurses during the COVID-19 lockdown and further explore the association of the strategies with nurses’ physical and mental health, family relationships, and job performance. We found five strategies: (1) the husband does it all, (2) fully outsourcing to grandparents, (3) partially outsourcing to grandparents, with the husband filling in the gap, (4) egalitarian remote workers, and (5) a neo-traditional strategy. Nurses who applied the egalitarian strategy generally had better physical and mental health and job performance, while those who applied the neo-traditional strategy had the least favorable outcomes, although they had the most sleep hours (by staying at home). The “husband does it all” strategy and the outsourcing strategies seemed to have mixed effects with high family cohesion, low relational tension, and high self-rated job performance, but also high psychological distress and fewer sleep hours.

Our findings on the work–family strategies among nurses during the lockdown period are different from previous research conducted during the pandemic. Shockley et al. found that during the pandemic, a large portion of dual-earner couples used highly gendered strategies, with wives working remotely and taking on most of the childcare, and little adjustment of husbands’ work roles [4]. Likewise, although nurses took on more work during the pandemic, they still played the main roles in the families [15,16,17]. However, in our study, most families adopted the reversed gender-asymmetrical strategy or non-traditional strategy (i.e., male partners performing all the childcare) and the strategy of fully outsourcing to grandparents. The use of a non-traditional strategy may be attributable to the fact that most nurses were required to work under a closed-loop management regime during the whole lockdown period and were not able to return home and fulfill their family roles [22,23,24,25]. Accordingly, their male partners had to take on more responsibilities of childcare and household management. Furthermore, when their husbands were also not available during the period, the couples had to outsource their family duties to their grandparents. This might be a temporal arrangement similar to Plagg et al., who found that during a week of “hard lockdown”, half of the Italian parents sought help from grandparents [29]. This strategy may also reflect traditional Chinese culture, which emphasizes grandparenting [43,44]. In other words, grandparents may have already taken on the major responsibility of childcare before the pandemic, which is a quite common phenomenon among dual-career Chinese couples who have young children [42,43].

Regarding the association of the work–family management strategies with individual and family outcomes, some of our findings were consistent with previous research. For example, Shockley et al. found that the “Wife Remote and Doing It All” strategy had the least favorable outcome with low family cohesion, high relational tension, and low job performance, and the egalitarian strategy of “Alternating Days” had the best outcomes [4]. Likewise, we found that the neo-traditional strategy was related to more family conflicts and lower job performance of nurses, while the egalitarian strategy was positively related to nurses’ physical and mental health and job performance. However, we also provided new evidence of the double-edged effects of the non-traditional strategy and the outsourcing strategy. On the one hand, a clear division of labor could reduce family conflicts and enhance family cohesion, which may also facilitate nurses’ job performance but, on the other hand, nurses who adopted these strategies also experienced less sleep hours and more psychological distress. Although past research suggests that men’s participation in housework and childcare is beneficial for women’s well-being [47], this may not be the case for a completely reversed gender-asymmetrical family role of nurses caused by closed-loop management and the lockdown policy. This role is contrary to the common/traditional expectations of women’s family roles [5,33] and may result in further role confusion and more guilt toward their families and children [52]. The situation may also be the same for husbands who were suddenly given the full responsibility of childcare. They may have also been highly confused and distressed [51,53] and transferred their negative feelings to their wives at work. Furthermore, although grandparenting can serve as a substitution for parenthood, it may still be considered “illegal” [29] and suffer from social stigma [43]. Children raised by grandparents may have more emotional and behavioral problems [43]. Grandparents also faced the challenges of managing the online learning of their grandchildren during the pandemic [65,66]. All these things may have become the major stressors for nurses.

This study makes three main contributions to knowledge. First, we classified the work–family management strategies adopted by nurses during the COVID-19 lockdown. Previous studies have paid attention to the mental health and family situations of nurses during the pandemic [1,2,3,13,14,15,16,17,18], but have not explored their work–family strategies under the extreme situation of lockdown and closed-loop management in hospitals. Second, unlike previous studies conducted in normal periods, we found that a reversed gender-asymmetrical strategy and the strategies of outsourcing to grandparents became more prevalent in nurse families during the extreme lockdown period, with women hardly participating in childcare and housework. Third, we explored the association of work–family management strategies with nurses’ physical and mental health, family relationships, and job performance and further revealed the potential double-edged effects of the non-traditional strategy and the strategy of fully outsourcing to grandparents. Those strategies may have increased the involvement of nurses in their work, which were also related to higher family cohesion and less relationship tension, but they were also related to more distress among nurses.

Regarding practical implications, because work–family management strategies play an important role in nurses’ health and performance, for future planning and risk mitigation purposes, practitioners and policymakers should pay attention to possibilities operating at a family level during the lockdown period. Rather than providing a one-size-fits-all solution [67] or a direct service of childcare [67], we recommend considering the diversity of the work–family strategies and providing tailored support. First, due to the prevalence of the “husband does it all” strategy and the outsourcing strategies, and their potential double-edged effects, nurses should be encouraged to discuss options with their family members before the lockdown in order to prepare for the changes in family roles and reduce possible role ambiguity and stress afterward. Husbands and grandparents should also be provided with the necessary help to facilitate their housework and childcare and be informed about the potential external resources (e.g., school teachers). Second, because of the positive association of the egalitarian “Alternating Days” strategy with health and job performance, policymakers should provide regular home-return opportunities for medical personnel and reduce obstacles to returning home as much as possible. An alternative arrangement could be better for nurses’ mental health than a totally closed-loop management. Finally, although there was only a small percentage of nurses staying at home for the whole period (possibly due to the early lockdown of their communities), their low family cohesion and high relational tension should be noted. Hospitals should establish multidisciplinary mental health promotion teams [1], communicate with their staff at home on a regular basis, and provide counseling for family affairs if necessary.

This study is not without limitations. First, our data were mainly cross-sectional and based on self-reported recall. It is well-established that longitudinal evidence can be more effective in uncovering causal relationships associated with working life—e.g., [4]. Second, the association of the work–family management strategies with individual and family outcomes found in this study may be temporal and only valid during the lockdown period. For instance, the potential double-edged effect of the non-traditional strategy and the full outsourcing strategy may have been due to the role confusion and stress as a result of the sudden lockdown. Third, some findings (e.g., fully outsourcing to grandparents) may be culturally specific. As such, we see considerable merit in testing the generalizability of our findings in other countries.

## 6. Conclusions

This study identified five work–family strategies among nurses during the COVID-19 lockdown period: (1) fully outsourcing to grandparents, (2) partially outsourcing to grandparents, with the husband filling in the gap, (3) the husband does it all, (4) egalitarian remote workers, and (5) a neo-traditional strategy. Nurses who applied the egalitarian strategy had better individual health and performance, and less relationship tension, while those who used the neo-traditional strategy had the least favorable outcomes. The gender-asymmetric and outsourcing strategies seem to have double-edged effects, with better job performance and family cohesion but also higher psychological distress and fewer sleeping hours among nurses. Due to the diversity of the strategies and their potential effects, health practitioners and policymakers should provide tailored support to nurses and their families during a future public health crisis.

## Figures and Tables

**Table 1:** Description of the categorical variables associated with work–family management strategies (*n* = 287).

Categorical Variables	Frequency (%)	
Remote Work States of Nurses		
Not working remotely (working in hospitals during the whole lockdown)	134 (46.7%)	
Working remotely partially (stable schedule, 5 days in hospitals and 2 days at home)	73 (25.4%)	
Mostly working remotely (working in hospitals at the beginning and then staying at home)	59 (20.6%)	
Working remotely full time (staying at home during the whole lockdown)	21 (7.3%)	
Outside Help		
Full-time help from the elders (e.g., grandparents)	173 (60.3%)	
No outside help	69 (24.0%)	
Part-time help from the elders (e.g., grandparents)	31 (10.8%)	
Relative, friend, or older sibling helping part-time	4 (1.4%)	
Paid help (e.g., nanny or babysitter) full-time	4 (1.4%)	
Relative, friend, or older sibling helping full-time	3 (1.0%)	
Paid help (e.g., nanny or babysitter) part-time	1 (0.3%)	
Relative/friend helping part-time and paid help part-time	1 (0.3%)	
Part-time help from community social workers or volunteers	1 (0.3%)	
Shift Change	Wives	Husbands
Informally changing a shift	117 (40.8%)	93 (32.4%)
Formally changing a shift	101 (35.2%)	102 (35.5%)
Not changing shift	69 (24.0%)	92 (32.1%)
Amount of Work Hour Change		
Increasing hours	174 (60.6%)	100 (34.8%)
No adjustment of hours	80 (27.9%)	63 (22%)
Reducing hours	33 (11.5%)	124 (43.2%)
Division of Childcare Labor during Normal Work Hours		
Performing none of the childcare	73 (25.4%)	15 (5.2%)
Full outsourcing	53 (18.5%)	53 (18.5%)
Only providing occasional childcare relief	44 (15.3%)	42 (14.6%)
Providing most of the childcare except occasional relief	29 (10.1%)	51 (17.8%)
Alternating working days with spouse	28 (9.8%)	28 (9.8%)
Staggering shift work	25 (8.7%)	23 (8.0%)
Performing all childcare during work hours	17 (5.9%)	58 (20.2%)
Performing all childcare when it is not outsourced	11 (3.8%)	7 (2.4%)
Both remote, alternating watching child with spouse based on meetings	4 (1.4%)	6 (2.1%)
Having mini-shifts throughout day while both are remote	3 (1.0%)	4 (1.4%)

**Table 2:** The means, standard deviations, and correlations of control and outcome variables (*n* = 287).

	*M*	*SD*	1	2	3	4	5	6	7	8	9	10	11
1. Nurses’ age	36.04	4.43	-										
2. Husbands’ age	38.19	5.22	0.77	-									
3. Age difference of couples	2.15	3.32	−0.12	0.54	-								
4. Household income	4.11	1.47	0.19	0.18	0.04	-							
5. No. of children < Grade 5	1.11	0.32	−0.04	0.00	0.04	0.05	-						
6. No. of children < 18 years	1.22	0.50	0.15	0.17	0.07	0.06	0.56	-					
7. Family cohesion	13.56	2.43	−0.03	−0.04	−0.02	0.05	0.02	−0.08	-				
8. Relationship tension	6.05	2.87	−0.10	−0.03	0.10	−0.09	0.00	0.00	−0.31	-			
9. Sleep hours	6.39	1.07	−0.07	−0.12	−0.11	0.03	0.03	−0.04	0.04	0.06	-		
10. Psychological distress	28.82	9.03	−0.02	0.00	0.02	−0.08	−0.11	0.03	−0.16	0.30	−0.25	-	
11. General job performance	16.70	2.77	0.06	0.06	0.01	0.10	0.01	0.00	0.29	−0.27	−0.17	−0.14	-
12. Relative job performance	74.39	27.63	−0.02	0.04	0.09	0.01	0.01	−0.10	0.08	−0.04	0.04	−0.14	0.18

Note: All the correlation coefficients in bold are significant. Those equaling 0.12 or more are significant at level of 0.05, and those equaling 0.16 or more are significant at level of 0.01.

**Table 3:** LCA fit statistics (*n* = 287).

No. of Classes	LL	FP	AIC	C-AIC	BIC	SSA-BIC	LMR (*p*)	BLRT (*p*)	Entropy
class 1	−3023.69	37	6121.38	6158.38	6256.78	6139.45	N/A	N/A	N/A
class 2	−2841.07	75	5832.14	5907.14	6106.6	5868.77	<0.001	<0.001	1
class 3	−2724.07	113	5674.13	5787.13	6087.66	5729.32	0.76	<0.001	0.985
class 4	−2658.54	151	5619.08	5770.08	6171.66	5692.82	0.44	<0.001	0.933
class 5	−2616.67	189	5611.34	5800.34	6302.99	5703.65	0.79	<0.001	0.938
class 6	−2587.92	227	5629.84	5856.84	6460.54	5740.7	0.76	0.21	0.93
class 7	−2564.45	265	5658.89	5923.89	6628.66	5788.31	0.85	0.43	0.929
class 8	−2541.31	303	5688.62	5991.62	6797.44	5836.6	0.79	0.8	0.947

Note: LCA = Latent Class Analysis. LL = log likelihood. FP = free parameters. AIC = Akaike information criterion. C-AIC = consistent Akaike information criterion. BIC = Bayesian information criterion. SSA-BIC = sample-size-adjusted Bayesian information criterion. LMR = Lo–Mendell–Rubin likelihood ratio test. BLRT = bootstrapped log-likelihood ratio test.

**Table 4:** Work–family management strategies of nurses during the COVID-19 lockdown (*n* = 287).

Strategy	Remote Work State (Nurse)	Outside Help	Shift Change		Amount of Hours Change		Division of Childcare Labor	
			Nurse	Husband	Nurse	Husband	Nurse	Husband
Husband does it all (29.3%)	Not working remotely (69%)	Full-time help from the elders (48.8%)	Not changing (44.0%)	Not changing (56.0%)	Increasing (61.9%)	Reducing (53.6%)	Performing none of the childcare (60.7%)	Performing all childcare during work hours (69.0%)
Fully or partially outsourcing to grandparents and husband filling in gap (25.8%)	Not working remotely (40.5%)	Full-time help from the elders (70.3%)	Informally changing (55.4%)	Informally changing (47.3%)	Increasing (68.9%)	Reducing (43.2%)	Only providing occasional childcare relief (52.7%)	Performing most of the childcare except occasional relief (40.5%)
Egalitarian remote workers with alternation of work days and hours (20.6%)	Mostly working remotely (45.8%) ^a^	No outside help (42.4%)	Informally changing (49.2%)	Informally changing (44.1%)	Increasing (47.5%)	Reducing (54.2%)	Alternating working days with spouse (44.1%)	Alternating working days with spouse (44.1%)
Fully outsourcing to grandparents (18.5%)	Not working remotely (64.2%)	Full-time help from the elders (90.6%)	Formally changing (47.2%)	Formally changing (43.4%)	Increasing (71.7%)	Increasing (67.9%)	Full outsourcing (100%)	Full outsourcing (100%)
Neo-traditional strategy (5.9%)	Mostly working remotely (29.4%)	Full-time help from the elders (41.2%)	Not changing (58.8%)	Formally changing (58.8%)	No adjustment (41.2%)	Increasing (47.1%)	Performing all childcare when it is not outsourced (58.8%)	Performing none of the childcare (47.1%)

Note: For interpretive purposes, the values in parentheses represent the option with the highest percentage in each categorical variable. ^a^ The percentage of “working remote partially” was also high (37.3%) in the egalitarian strategy.

**Table 5:** Mean differences of work–family management strategies in nurses’ physical and mental health, family relationships, and job performance (*n* = 287).

	Husband Does it all (A)	Fully or Partially Outsourcing to Grandparents and Husband Filling in Gap (B)	Egalitarian Remote Workers with Alternation of Work Days and Hours (C)	Fully Outsourcing to Grandparents (D)	Neo-Traditional Strategy (E)	Overall Chi-Square
1. Family cohesion	13.40	13.72	13.17D	14.19C	13.00	8.45 †
2. Relationship tension	5.95E	5.96E	5.95E	5.98E	7.41ABCD	5.98
3. Sleep hours	6.37	6.32	6.56D	6.17CE	6.94D	9.50 *
4. Psychological distress	30.51C	27.97	26.10AD	30.53C	28.18	14.63 **
5. General job performance	16.50	17.15E	16.68E	16.85E	15.35BCD	8.04 †
6. Relative job performance	76.74	72.14	75.15	71.89	77.82	1.86

Note: * *p* < 0.05. ** *p* < 0.01. † *p* < 0.1. The letters represent the mean of the strategy was statistically significantly different from that of the current strategy (*p* < 0.05).

## Data Availability

The data are available from the corresponding author upon request.
